# Guanylate-Binding Proteins Are Critical for Effective Control of *Francisella tularensis* Strains in a Mouse Co-Culture System of Adaptive Immunity

**DOI:** 10.3389/fcimb.2020.594063

**Published:** 2020-12-10

**Authors:** Nasibeh Mohammadi, Helena Lindgren, Igor Golovliov, Kjell Eneslätt, Masahiro Yamamoto, Amandine Martin, Thomas Henry, Anders Sjöstedt

**Affiliations:** ^1^ Department of Clinical Microbiology and Laboratory for Molecular Infection Medicine Sweden (MIMS), Umeå University, Umeå, Sweden; ^2^ Department of Immunoparasitology, Research Institute for Microbial Diseases, Osaka, Japan; ^3^ CIRI, Centre International de Recherche en Infectiologie, Inserm U1111, Université Claude Bernard Lyon 1, CNRS, UMR5308, ENS de Lyon, Univ Lyon, Lyon, France

**Keywords:** *Francisella tularensis*, guanylate-binding proteins, mouse co-culture model, cytokine patterns, correlates of protection

## Abstract

*Francisella tularensis* is a Select Agent that causes the severe disease tularemia in humans and many animal species. The bacterium demonstrates rapid intracellular replication, however, macrophages can control its replication if primed and activation with IFN-γ is known to be essential, although alone not sufficient, to mediate such control. To further investigate the mechanisms that control intracellular *F. tularensis* replication, an *in vitro* co-culture system was utilized containing splenocytes obtained from naïve or immunized C57BL/6 mice as effectors and infected bone marrow-derived wild-type or chromosome-3-deficient guanylate-binding protein (GBP)-deficient macrophages. Cells were infected either with the *F. tularensis* live vaccine strain (LVS), the highly virulent SCHU S4 strain, or the surrogate for *F. tularensis*, *F. novicida*. Regardless of strain, significant control of the bacterial replication was observed in co-cultures with wild-type macrophages and immune splenocytes, but not in cultures with immune splenocytes and *GBP* ^chr3^-deficient macrophages. Supernatants demonstrated very distinct, infectious agent-dependent patterns of 23 cytokines, whereas the cytokine patterns were only marginally affected by the presence or absence of GBPs. Levels of a majority of cytokines were inversely correlated to the degree of control of the SCHU S4 and LVS infections, but this was not the case for the *F. novicida* infection. Collectively, the co-culture assay based on immune mouse-derived splenocytes identified a dominant role of GBPs for the control of intracellular replication of various *F. tularensis* strains, regardless of their virulence, whereas the cytokine patterns markedly were dependent on the infectious agents, but less so on GBPs.

## Introduction

*Francisella tularensis* is a facultative intracellular bacterium and the etiological agent of tularemia, a highly infectious disease affecting humans and a wide range of animals. *F. tularensis* is highly contagious, since its infectious dose is very low and it may be spread *via* aerosol. These characteristics, together with high virulence, have led to its classification as a Tier 1 Select Agent, along with other potential agents of bioterrorism. Tularemia is an emerging disease in several parts of the world and extensive outbreaks have occurred in some countries in Scandinavia and Eastern Europe and in Turkey. Two subspecies cause human disease, subspecies *tularensis*, with high mortality resulting if untreated, and subspecies *holarctica*, which despite lower virulence still can cause serious illness in humans. Currently, no licensed tularemia vaccine is available; however, the live vaccine strain (LVS) of subsp. *holarctica* has been used as a human vaccine and confers efficacious protection against laboratory-acquired infection (Burke, 1977). The closely related species *F. novicida* is an often used surrogate for *F. tularensis* and although a very rare human pathogen, it is virulent for many animal species and highly virulent in the frequently used mouse model of tularemia.

Cell-mediated immunity is critically required to control tularemia in human and animal models of the disease and therefore a comprehensive understanding of the protective cell-mediated mechanisms will be essential as part of the efforts to generate efficacious vaccines. Development of a licensed vaccine is dependent on the identification of correlates of protection; however, such correlates are elusive for infections requiring cell-mediated protection. With regard to tularemia, much work in experimental models have focused on the role of IFN-γ, which has been shown to be essential, although alone not sufficient to control infection. Population-based testing of vaccine candidates cannot be performed, since tularemia generally is an uncommon disease and even in endemic areas, it occurs highly irregularly (Sjöstedt, 2007) and, therefore, assessment of efficacy will not be feasible. Vaccine efficacy was evaluated in the 1950s by challenging volunteers with *F. tularensis*, however, it is unlikely that such future studies will be deemed ethically acceptable due to the severity of respiratory tularemia. To circumvent these limitations, an option is to utilize the FDA Animal Rule (Snoy, 2010) in order to license a tularemia vaccine. The rule is based on the premise of the exclusive use of relevant animal models to identify correlates of protection pertinent to human infection and thereafter to extrapolate data on efficacy in these models to efficacy in humans.

The existing evidence indicates that protection to *F. tularensis* is achieved through an intricate interaction of several T cells subsets and multiple cytokines that jointly effectuate control of infection (Cowley and Elkins, 2011; de Pascalis et al., 2012; Eneslätt et al., 2012; Mahawar et al., 2013; de Pascalis et al., 2014; Griffin et al., 2015; Golovliov et al., 2016). Thus, the mechanisms cannot be identified by use of infection assays based on a single cell type, rather assays are required that faithfully mirror the complex interplay that occurs *in vivo*. Therefore, models are required that allow detailed characterization of mechanisms that control bacterial intracellular replication and that also can be used to validate potential protective cell-mediated correlates, as required by the Animal Rule. Of relevance, a co-culture assay has been widely used for this purpose and is based on a combination of effector cells derived from naïve or immune animals that are added to cultures with infected monocytic cells (Elkins et al., 2011). Thereby, replication of *F. tularensis* can be followed over time and immune activation carefully assessed. There are numerous examples when the assay has been used to identify potential correlates of protection against *F. tularensis* (Collazo et al., 2009; Elkins et al., 2011; de Pascalis et al., 2012; Mahawar et al., 2013; de Pascalis et al., 2014; Griffin et al., 2015; Golovliov et al., 2016; de Pascalis et al., 2018; Eneslätt et al., 2018; Lindgren et al., 2020). To further assess the relevance of the findings, validation can be achieved by demonstrating that potential correlates identified contribute to protection in animal models (Kurtz et al., 2013; Melillo et al., 2013; Melillo et al., 2014). There are several animal models that are considered to be highly relevant as experimental models for tularemia, *e.g*., the mouse, rat, rabbit, and non-human primate models, since the target organs and histo-pathology closely resemble those of humans and all of these species can be naturally infected with *F. tularensis* (Lyons and Wu, 2007).

Many pathogenic bacteria and parasites have evolved sophisticated means to invade and replicate within host cells and in parallel, eukaryotes have developed counter-measures to effectively detect the invasion by these intracellular microorganisms and rapidly mount an antimicrobial response. One recently identified key mechanism for this detection is mediated by the Guanylate Binding Proteins (GBPs), belonging to a family of interferon-inducible dynamin-like GTPases. In fact, for many pathogenic intracellular bacteria and parasites, the role of GBPs appear essential for the execution of the IFN-γ-induced protective immune response (Tretina et al., 2019). However, how the effectuation of their antimicrobial functions is mediated is not fully understood, but it has been suggested that GBPs directly target and disrupt pathogen-containing vacuoles, as evidenced by findings on *Toxoplasma* and *Salmonella* (Man et al., 2017). In the case of Gram-negative bacteria, it has been observed that GBPs destabilize the rigidity of the bacterial outer membrane by binding to LPS and induce LPS clustering through GBP polymerization (Kutsch et al., 2020). There is also evidence for a link between GBPs and the inflammasomes (Meunier and Broz, 2016) and this has been clearly demonstrated to be the case for the AIM2 inflammasome in macrophages infected with *F. novicida* (Fernandes-Alnemri et al., 2010; Jones et al., 2010). In fact, the GBPs have been suggested to serve as master regulators of numerous inflammasomes (Wallet et al., 2017). After being ingested, *Francisella* strains rapidly lyse the phagosome, escape into and replicate within the host cytosol. In the murine system, the chromosome 3-encoded GBPs, GBP2 and GBP5, are recruited to cytosolic *F. novicida* and are required to lyse bacteria and release the bacterial genomic DNA into the host cytosol where it is recognized by AIM2 (Fernandes-Alnemri et al., 2010). However, although GBPs serve a key role for control of certain inflammasomes, control of intracellular *F*. *novicida* replication occurs independently of inflammasomes, but strictly dependent on GBPs (Wallet et al., 2017). Notably, *GBP* ^chr3^-deficient mice showed no control of an *F. novicida* infection, despite high levels of circulating IFN-γ (Wallet et al., 2017). Whereas control of intracellular replication of *F. novicida* and the LVS strain was completely reversed in the absence of GBPs, no control of the highly virulent SCHU S4 strain was observed, neither was exacerbation seen in the absence of GBPs (Wallet et al., 2017).

Here, we asked whether a complex co-culture system would provide additional information regarding the role of GBPs for the control of intracellular *F. tularensis* infection beyond what has already been revealed by the use of intramacrophage assays and the mouse model. As effector cells, Δ*clpB*-immune splenocytes were used, since they execute very potent control of *F. tularensis* infection (Golovliov et al., 2016). Cultures also contained bone marrow-derived macrophages (BMDM) from wild-type or *GBP* ^chr3^-deficient macrophages infected with either *F. novicida*, the LVS, or the SCHU S4 strain, the latter a highly virulent subspecies *tularensis* strain. We observed that in the co-culture system, in contrast to what is observed when cultures with BMDM alone, SCHU S4 replication was significantly controlled. Although control of SCHU S4 replication was not as marked as control of LVS or *F. novicida* replication, it was mostly GBP-dependent. Thus, the co-culture assay with immune mouse-derived cells identified a critical role of GBPs for the control of intracellular replication of various *F. tularensis* strains, regardless of their virulence.

## Materials and Methods

### Bacterial Strains

*F. tularensis* LVS (*F. tularensis* subsp. *holarctica*), *F. novicida* U112, and *F. tularensis* strain SCHU S4 (*F. tularensis* subsp. *tularensis*) were obtained from the American Type Culture Collection, ATCC 29684 and ATCC 15482 and from the *Francisella* Strain Collection of the Swedish Defense Research Agency, Umeå, Sweden, respectively. Work with the SCHU S4 strain was performed in a biosafety level 3 facility certified by the Swedish Work Environment Authority. The generation of the Δ*clpB* mutant and its utility as an efficacious vaccine have previously been described (Conlan et al., 2010; Golovliov et al., 2013).

### Animals

C57/BL6 mice obtained from Charles River, Germany were used. Immunization was performed with a subcutaneous injection of 5 × 10^3^ CFU of the Δ*clpB* strain. This results in an infection with no or only slight symptoms during peak replication of bacteria that occurs around day 4–6 of infection. *GBP^chr3–/–^* C57BL/6 mice have been previously described (Yamamoto et al., 2012). Ethical approval for the described mouse experiments was obtained from the Ethical Committee on Animal Research, Umeå, Sweden, A67-14 and A36-2019, or the University of Lyon, France (CEC-CAPP) under the protocol no. #ENS_2012_061 in accordance with the European regulations (#2010/63/UE).

### Generation of BMDM

Bone-marrow-derived macrophages were prepared by collecting bone marrow from the femurs of mice and then plating the cells in Petri dishes in DMEM (Invitrogen Life Technologies) and supplemented with 10% fetal calf serum (Invitrogen Life Technologies), 10 mM HEPES (Invitrogen Life Technologies), and 30% macrophage colony-stimulating factor (M-CSF)-conditioned medium. The latter was collected from an L929 M-CSF cell line. After incubation at 37°C in 5% CO_2_ for 6 days, BMDM were harvested and added to 24-well plates, incubated overnight, and then used in the co-culture assay. The viability of the BMDM was determined using staining with trypan blue and enumeration with a Vi-CELL XR cell viability analyzer (Beckman Coulter).

### Splenocyte Preparation

After immunization of mice, spleens were removed 4 to 5 weeks later and cells were obtained by squeezing the organ. They were prepared as previously described (Golovliov et al., 2016). The cell suspension was treated with ammonium chloride to lyse erythrocytes, washed with PBS + 2% FBS, and suspended in complete DMEM (cDMEM), DMEM supplemented with fetal calf serum and HEPES.

### Infection of the BMDM in the Co-Culture Assay

Bacteria were cultivated overnight on modified Gc-agar plates. After harvesting, they were added to BMDM cultures using an MOI of 0.2 of bacteria per BMDM and after 2 h, medium was removed and the cultures washed twice with DMEM. cDMEM with 20 µg/ml of gentamicin was added and after incubation for 45 min, cultures were washed with PBS. Then, 200 µl of cDMEM with 2.5 × 10^6^ splenocytes was added, resulting in a ratio of five splenocytes per BMDM. This was defined as time 0 h. After lysis of cells at 0 and 72 h, enumeration of bacteria was performed by plating of serial dilutions on Gc-agar plates.

### Cytokine Analysis

Supernatants from cell cultures were collected and stored at −80°C. Analysis was performed with a 23-plex kit (#M60009RDPD (BioRad Laboratories Inc, Hercules, CA, USA) using a Bio-Plex 200 system following the instructions of the manufacturer. Preliminary experiments indicated that significant increases of cytokine levels occurred between 24 and 48 h after infection, whereas changes between 48 and 72 h were mostly non-significant, therefore, the 48 h time point was chosen for analysis of cytokines.

### Data Analysis and Statistical Methods

All statistical analyses were performed using IBM SPSS statistics version 25. One-way ANOVA with Tukey’s test for multiple comparisons was used to analyze the significance of differences between different groups. A value of *P* < 0.05 was considered significant. Correlations were estimated using Spearman’s correlation. Twenty-three cytokines measured in co-cultures incubated with immune cells were analyzed by stepwise linear discriminant analysis (LDA) with Wilks’ lambda variable selection method. The F entry value was set to F > 3.84 and F removal value was set to F < 2.71.

## Results

### Intracellular Replication of *F. tularensis* Strains

**Figure 1:**
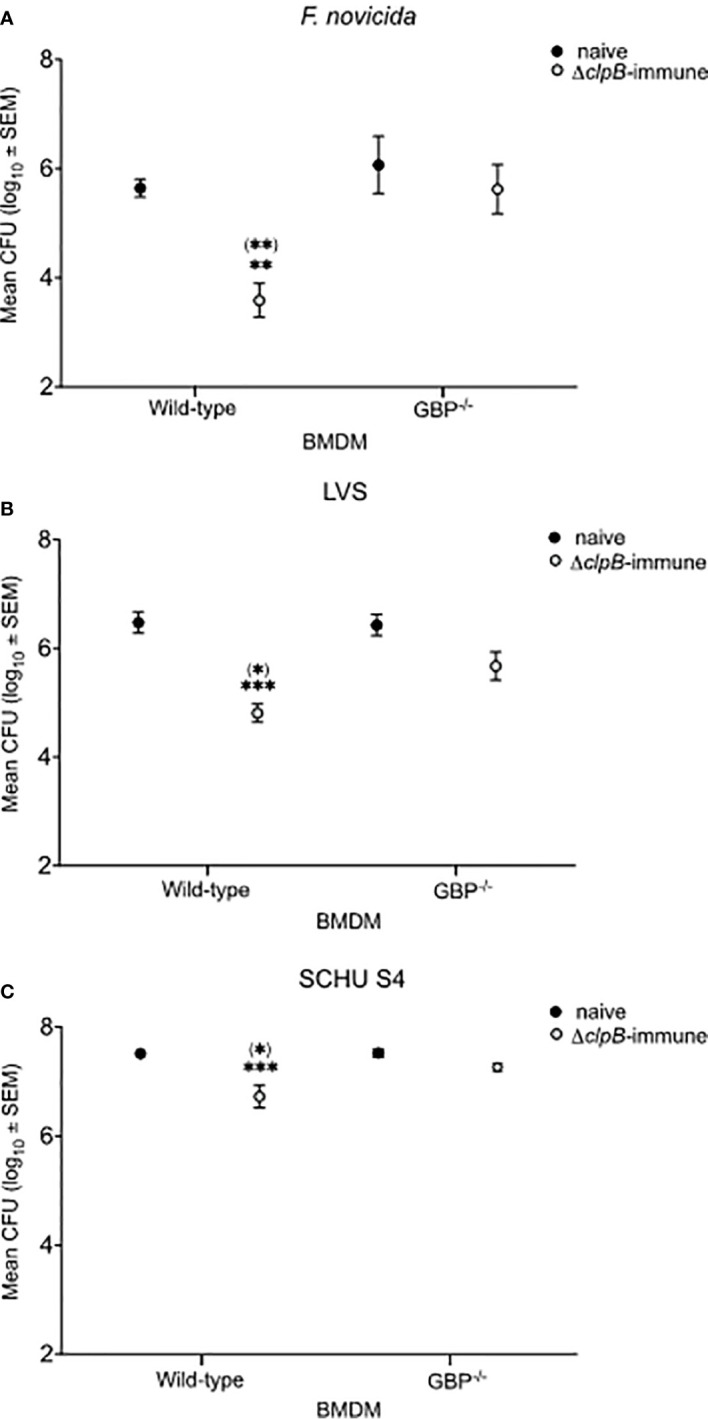
Intracellular replication of *F. tularensis* strains in the co-culture assay. BMDM monolayers were infected with **(A)** *F. novicida*, **(B)** LVS, or **(C)** SCHU S4 and thereafter overlaid with splenocytes from either naïve or Δ*clp*B-immunized mice. Bacterial numbers were recorded after 72 h of infection. The bars represent CFU values from nine cultures generated in three separate experiments. Stars above the bars indicate significant difference in comparison to the values of cultures with wild-type BMDM and naïve splenocytes. Stars in brackets indicate significant differences *vs*. cultures with *GBP* ^chr3−/−^-deficient BMDM with Δ*clp*B-immune splenocytes.

Co-cultures were established by overlaying BMDM monolayers infected with either the *F. novicida*, LVS, or SCHU S4 strains with splenocytes from naïve or Δ*clpB*-immune mice and bacterial replication was followed for a period of 72 h. In cultures with wild-type BMDM, the outcome of the *F. novicida* and LVS infections was rather similar and addition of immune splenocytes resulted in approximately 1.7 log_10_ CFU lower numbers than in cultures with naïve splenocytes (*P* < 0.01 for *F. novicida* and *P* < 0.001 for LVS; **Figures 1A, B**). In contrast, control of the SCHU S4 infection was less marked, approximately 0.7 log_10_ CFU lower in cultures with immune splenocytes (*P* < 0.01; **Figure 1C**). Regardless of infecting strain, bacterial replication was similar in cultures with naïve splenocytes and wild-type BMDM or *GBP* ^chr3^-deficient BMDM (*P* > 0.05; **Figures 1A–C**). Compared to cultures with wild-type BMDM, the CFU of the *GBP* ^chr3^-deficient BMDM cultures showed much smaller differences between immune and naïve splenocytes; 0.5 log_10_ CFU for *F. novicida*, 0.6 log_10_ CFU for LVS, and 0.2 log_10_ CFU for SCHU S4 (*P* > 0.05 for all strains; **Figures 1A–C**). Addition of immune splenocytes resulted in significantly lower bacterial numbers, regardless of infection, in cultures with wild-type BMDM than with *GBP* ^chr3^-deficient BMDM (*P* < 0.05 for *F. novicida* and SCHU S4 and *P* < 0.01 for LVS; **Figures 1A–C**).

### Cytokine Production in Co-Cultures

The levels of 23 cytokines were measured in the culture supernatants after 48 h of infection. A comparative analysis of the levels was performed and the results were expressed as *P*-values in **Tables 1** **–** **3**, with levels of the cultures infected with *F. novicida* as the reference, and the absolute values are provided in **Figure S1**.

**Table 1:** Differences in cytokine levels, expressed as *P*-values, between groups of *F. novicida*-infected co-cultures.

Cytokine/chemokine	Immune wild-type^1^/Naïve wild-type	*F. novicida* Immune GBP^−/−^/Naïve GBP^−/−^	Immune wild-type/Immune GBP^−/−^
IL-1α	0.923^2^	0.698	0.432
IL-1β	0.323	0.959	0.450
IL-2	<0.001	<0.001	0.211
IL-3	0.583	0.006	0.032
IL-4	0.009	<0.001	0.669
IL-5	0.991	0.994	0.324
IL-6	1.000	0.999	0.114
IL-9	0.996	0.981	0.725
IL-10	0.996	0.978	0.340
IL-12p40	0.833	0.103	0.096
IL-12p70	0.942	0.772	0.490
IL-13	0.995	0.837	0.384
IL-17	0.931	0.347	0.919
Eotaxin	0.913	0.967	0.390
G-CSF	1.000	0.132	0.960
GM-CSF	0.068	0.242	1.000
IFN-γ	0.546	0.008	0.080
KC	0.998	0.116	0.592
MCP-1	0.785	1.000	0.023
MIP-1α	0.869	0.017	0.989
MIP-1β	0.319	0.550	0.809
RANTES	0.636	0.098	0.521
TNF	1.000	0.425	0.886

^1^Immune means that splenocytes from clpB-immunized had been added to the co-cultures and naive means that naive splenocytes from non-immunized mice had been added. Wild-type means that co-cultures contained BMDM from wild-type mice and GBP^−/−^ means that BMDM had been obtained from GBP^chr3−/−^-deficient mice.

^2^P-values are indicated for the comparison between cytokine levels in the group indicated as numerator vs. the denominator according to one-way ANOVA with Tukey Post-Hoc test.

^3^Red color indicates that the numerator is significantly higher (P < 0.05) than the denominator.

^4^Blue color indicates that the denominator is significantly higher (P < 0.05) than the numerator.

In cultures infected with *F. novicida*, IL-2 was the only cytokine expressed at significantly higher levels in wild-type BMDM cultures with immune *vs*. naïve splenocytes, whereas levels of IL-4 was higher in the latter (**Table 1**). In cultures with *GBP* ^chr3^-deficient BMDM, again, IL-2, together with IL-3, were higher in cultures with immune *vs*. naïve splenocytes and, again, IL-4, as well as MIP-1β and IFN-γ, were higher in the latter cultures (**Table 1**). When cultures with immune splenocytes were compared, IL-3 was the sole cytokine expressed at higher levels in cultures with wild-type BMDM and MCP-1 the only cytokine expressed at higher levels with *GBP* ^chr3^-deficient BMDM.

After infection with LVS, six cytokines were significantly higher in wild-type BMDM cultures with immune *vs*. naïve splenocytes, whereas in cultures with GBP-deficient BMDM IL-2 and IL-3 were higher and IL-4 lower with immune *vs*. naïve splenocytes (**Table 2**). When cultures with immune splenocytes were compared, levels of five cytokines, IL-5, eotaxin, MCP-1, MIP-1β, and RANTES, were higher with in cultures with wild-type BMDM *vs*. *GBP* ^chr3^-deficient BMDM (**Table 2**).

**Table 2:** Differences, expressed as *P*-values, in cytokine levels between groups of LVS-infected co-cultures.

Cytokine/chemokine	Immune wild-type^1^/Naïve wild-type	LVS Immune GBP^−/−^/Naïve GBP^−/−^	Immune wild-type/Immune GBP^−/−^
IL-1α	0.070^2^	0.269	0.658
IL-1β	0.067	0.970	0.102
IL-2	0.032	0.008	0.979
IL-3	0.038	0.044	0.999
IL-4	0.559	0.007	0.899
IL-5	0.062	0.751	0.005
IL-6	0.974	0.962	0.692
IL-9	0.176	0.254	0.111
IL-10	0.589	0.439	0.973
IL-12p40	0.983	0.990	0.805
IL-12p70	0.759	0.118	0.724
IL-13	0.154	0.079	0.256
IL-17	0.038	0.069	0.975
Eotaxin	0.414	0.598	0.031
G-CSF	0.834	0.918	0.987
GM-CSF	0.005	0.390	0.093
IFN-γ	0.033	0.228	0.830
KC	0.769	0.601	0.621
MCP-1	0.488	0.998	0.021
MIP-1α	0.930	0.379	0.578
MIP-1β	0.913	0.315	0.036
RANTES	0.003	0.826	0.024
TNF	0.071	0.110	0.942

^1^Immune means that splenocytes from clpB-immunized had been added to the co-cultures and naive means that naive splenocytes from non-immunized mice had been added. Wild-type means that co-cultures contained BMDM from wild-type mice and GBP^−/−^ means that BMDM had been obtained from GBP^chr3−/−^-deficient mice.

^2^P-values are indicated for the comparison between cytokine levels in the group indicated as numerator vs. the denominator according to one-way ANOVA with Tukey Post-Hoc test.

^3^Red color indicates that the numerator is significantly higher (P < 0.05) than the denominator.

^4^Blue color indicates that the denominator is significantly higher (P < 0.05) than the numerator.

After infection with SCHU S4, 18 cytokines were secreted at significantly higher levels in wild-type BMDM cultures with immune *vs*. naïve splenocytes, whereas in GBP-deficient cultures, 20 cytokines were higher with immune *vs*. naïve splenocytes (**Table 3**). Eight cytokines were expressed at higher levels in immune splenocyte co-cultures with wild-type BMDM *vs*. co-cultures with *GBP* ^chr3^-deficient BMDM, including IL-1β, MCP-1, MIP-1β, IL-12p40, and RANTES, whereas two, IL-1α and KC, were higher in the latter cultures (**Table 3**). These results demonstrate that the immune splenocytes induced a pronounced immune activation in co-cultures regardless of the BMDM phenotype and also, in comparison to LVS- or *F. novicida*-infected cultures, that the differences between cultures with immune *vs.* naïve splenocytes were much more marked for the SCHU S4-infected cultures.

**Table 3:** Differences, expressed as *P*-values, in cytokine levels between groups of SCHU S4-infected co-cultures.

Cytokine/chemokine	Immune wild-type^1^/Naïve wild-type	SCHU S4 Immune GBP^−/−^/Naïve GBP^−/−^	Immune wild-type/Immune GBP^−/−^
IL-1α	0.013^2^	<0.001	0.007
IL-1β	<0.001	<0.001	<0.001
IL-2	0.001	<0.001	0.871
IL-3	<0.001	<0.001	0.834
IL-4	0.488	0.008	0.358
IL-5	<0.001	0.008	<0.001
IL-6	0.004	<0.001	0.845
IL-9	0.001	<0.001	0.014
IL-10	0.013	<0.001	0.999
IL-12p40	<0.001	0.005	0.003
IL-12p70	0.022	<0.001	1.000
IL-13	0.001	0.001	0.060
IL-17	0.144	0.009	1.000
Eotaxin	<0.001	<0.001	<0.001
G-CSF	0.040	<0.001	0.997
GM-CSF	<0.001	0.001	0.161
IFN-γ	0.006	0.006	1.000
KC	0.417	<0.001	<0.001
MCP-1	<0.001	0.306	<0.001
MIP-1α	0.887	0.003	0.347
MIP-1β	0.259	0.031	0.006
RANTES	0.003	0.744	0.021
TNF	<0.001	<0.001	0.080

^1^Immune means that splenocytes from clpB-immunized had been added to the co-cultures and naive means that naive splenocytes from non-immunized mice had been added. Wild-type means that co-cultures contained BMDM from wild-type mice and GBP^-/-^ means that BMDM had been obtained from GBP^chr3-/–^deficient mice.

^2^P-values are indicated for the comparison between cytokine levels in the group indicated as numerator vs. the denominator according to one-way ANOVA with Tukey Post-Hoc test.

^3^Red color indicates that the numerator is significantly higher (P < 0.05) than the denominator.

^4^Blue color indicates that the denominator is significantly higher (P < 0.05) than the numerator.

Relative cytokine levels in cultures with immune splenocytes are shown for all infections in **Tables 4** and **5**, with levels of the cultures infected with *F. novicida* as the reference, and the absolute values are provided in **Figure S1**. In cultures with immune splenocytes and wild-type macrophages, levels of a majority of cytokines were highest in those infected with SCHU S4. Compared to the *F. novicida*-infected cultures, levels of 15 cytokines were significantly higher, including IL-2, IFN-γ, TNF, GM-CSF, and IL-17 (**Table 4**). The cultures with the same cellular composition infected with LVS showed higher levels of eight cytokines compared to the *F. novicida*-infected cultures, including the aforementioned cytokines. A comparison between SCHU S4 and LVS-infected cultures, demonstrated that the levels were higher for 12 cytokines of the former and one of the latter. In cultures with immune splenocytes and *GBP* ^chr3^-deficient macrophages, the differences between the infections were not consistently higher in one or the other. When cultures infected with SCHU S4 were compared to those infected with *F. novicida*, levels were higher for IL-3, the Th2 cytokine IL-4 and the Th1 cytokine GM-CSF of the former and the chemokine RANTES was higher of the latter. The same comparison between LVS- and *F. novicida*-infected cultures, demonstrated higher levels of IL-1α and KC for the latter (**Table 5**).

**Table 4:** Analysis of cytokine levels in *F. novicida*-infected co-cultures with wild-type BMDM and immune splenocytes compared to the corresponding cultures infected with LVS or SCHU S4.

Cytokine	LVS^1^	SCHU S4^2^
IL-1α	Equal^3^	++^4^
IL-1β	Equal	++
IL-2	Equal	+
IL-3	Equal	+++
IL-4	+	++
IL-5	Equal	Equal
IL-6	Equal	Equal
IL-9	Equal	Equal
IL-10	+	+
IL-12p40	Equal	Equal
IL-12p70	Equal	++
IL-13	+	++
IL-17	Equal	+
Eotaxin	+	Equal
G-CSF	++	+ #70ad47+
GM-CSF	Equal	++
IFN-γ	Equal	Equal
KC	+	++
MCP-1	Equal	Equal
MIP-1α	Equal	+
MIP-1β	Equal	Equal
RANTES	+	+
TNF	+	++

^1^LVS-infected co-cultures with wild-type BMDM and immune splenocytes compared to the corresponding cultures infected with F. novicida.

^2^SCHU S4-infected co-cultures with wild-type BMDM and immune splenocytes compared to the corresponding cultures infected with F. novicida.

^3^Equal indicates that the cytokine levels do not significantly differ (P > 0.05) from those of the corresponding F. novicida infection.

^4^Green colors with +, ++, and +++ indicate that levels are higher, P < 0.05, P < 0.01, and P < 0.001 respectively, compared to the levels in the F. novicida cultures. P-values were determined using one-way ANOVA with Tukey HSD Post-Hoc test.

**Table 5:** Analysis of cytokine levels in *F. novicida*-infected co-cultures with *GBP* ^chr3−/−^-deficient BMDM and immune splenocytes compared to the corresponding cultures infected with LVS or SCHU S4.

Cytokine	LVS^1^	SCHU S4^2^
IL-1α	+^3^	Equal^4^
IL-1β	Equal	Equal
IL-2	Equal	Equal
IL-3	Equal	++^5^
IL-4	Equal	+
IL-5	Equal	Equal
IL-6	Equal	Equal
IL-9	Equal	Equal
IL-10	Equal	Equal
IL-12p40	Equal	Equal
IL-12p70	Equal	Equal
IL-13	Equal	Equal
IL-17	Equal	Equal
Eotaxin	Equal	Equal
G-CSF	Equal	Equal
GM-CSF	Equal	+++
IFN-γ	Equal	Equal
KC	+	Equal
MCP-1	Equal	Equal
MIP-1α	Equal	Equal
MIP-1β	Equal	Equal
RANTES	Equal	+
TNF	Equal	++

^1^LVS-infected co-cultures with GBP^chr3−/−^-deficient BMDM and immune splenocytes compared to the corresponding cultures infected with F. novicida.

^2^SCHU S4-infected co-cultures with GBP^chr3−/−^-deficient BMDM and immune splenocytes compared to the corresponding cultures infected with F. novicida.

^3^Red color with + indicates that levels are lower, P < 0.05, compared to the levels in the F. novicida cultures. P-values were determined using one-way ANOVA with Tukey HSD Post-Hoc test.

^4^Equal indicates that the cytokine levels do not significantly differ (P > 0.05) from those of the corresponding F. novicida infection.

^5^Green colors with +, ++, and +++ indicate that levels are higher, P < 0.05, P < 0.01, and P < 0.001 respectively, compared to the levels in the F. novicida cultures. P-values were determined using one-way ANOVA with Tukey HSD Post-Hoc test.

### Correlation Between Cytokines and Control of Bacterial Infection

Levels of individual cytokines were correlated to bacterial numbers after 72 h. A majority of cytokines in the SCHU S4- and LVS-infected cultures, 17 and 15, respectively, was significantly inversely correlated to bacterial numbers with Spearman´s rho of >0.5 (*P* < 0.05; **Table 6**). In contrast, none of the 23 cytokines showed an inverse correlation to bacterial numbers in the *F. novicida*-infected cultures, instead 13 showed positive correlation with bacterial numbers (**Table 6**). Thus, levels of many individual cytokines were inversely correlated with the bacterial numbers of the SCHU S4- and LVS-infected cultures, whereas a majority of cytokines showed a positive correlation with the numbers of *F. novicida*. Of the 17 cytokines in the SCHU S4 cultures and 15 cytokines in the LVS cultures that showed significant inverse correlations, several have previously been identified as correlates of protection, *e.g*., IL-2, IFN-γ, TNF, IL-12p40, GM-CSF, and IL-17 and most of these cytokines are also characteristic of a Th1 T cell response. Notably, IL-2, IFN-γ, IL-12p40, and GM-CSF were not among the 13 cytokines that correlated to bacterial numbers in the *F. novicida*-infected cultures.

**Table 6:** Correlations between cytokine and CFU levels for respective infection.

	SCHU S4	LVS	*F. novicida*
IL-1α	−0.56*	−0.41	0.75
IL-1β	−0.67	−0.54	0.70
IL-2	−0.72	−0.64	−0.07
IL-3	−0.66	−0.60	0.28
IL-4	−0.07	0.62	0.63
IL-5	−0.47	−0.66	0.24
IL-6	−0.65	−0.64	0.62
IL-9	−0.63	−0.69	0.41
IL-10	−0.74	−0.66	0.62
IL-12(p40)	−0.73	−0.75	0.17
IL-12(p70)	−0.57	−0.50	0.76
IL-13	−0.59	−0.46	0.59
IL-17	−0.66	−0.58	0.67
Eotaxin	−0.55	−0.58	0.27
G-CSF	−0.62	−0.47	0.68
GM-CSF	−0.73	−0.67	0.17
IFN-γ	−0.64	−0.61	0.10
KC	−0.41	−0.28	0.76
MCP-1	−0.39	−0.52	−0.03
MIP-1α	−0.42	−0.00	0.83
MIP-1β	−0.49	−0.41	0.51
RANTES	−0.69	−0.73	0.31
TNF	−0.68	−0.60	0.70

*Spearman’s correlation was determined. A value >0.50 indicates that P < 0.01.

Green color indicates that CFU values were inversely correlated (P < 0.01) to cytokine levels.

Red color indicates that CFU values were positively correlated (P < 0.01) to cytokine levels.

### Discrimination of Co-Cultures With Immune Cells Based on Cytokine Patterns

**Figure 2:**
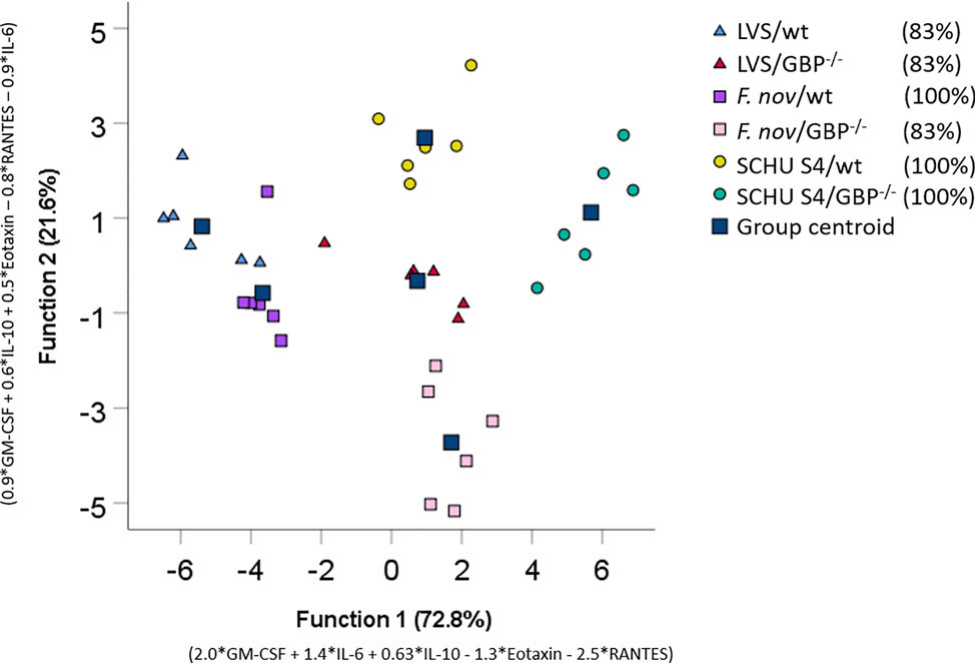
Discriminant loadings of co-cultures. Twenty-three cytokines measured in co-cultures incubated with immune cells were included in a stepwise discriminant function analysis. Function 1 and 2 explains 72.8 and 21.6% of the variance respectively and are depicted on the x- and y-respectively. Each data point corresponds to each replicate individual in a group and the squares represent the group centroid. The percentage values of group classification correctness are presented in brackets.

Linear discriminant analysis was used to determine whether individual cytokines, or sets of cytokines, could differentiate between the six groups of co-cultures with immune cells. The cytokines giving the best separation of the groups were GM-CSF, IL-6, IL-10, Eotaxin, and RANTES (**Table 7**). To further illustrate the discriminative ability of the set of cytokines, the data were plotted using discriminant loading (**Figure 2**). The results demonstrate that both the infectious agent and BMDM phenotype affected the location of each group (**Figure 2**). The SCHU S4-infected wild-type and *GBP* ^chr3^-deficient cultures were relatively distant from each other and also distinct from the other four groups. The LVS-infected cultures demonstrated an intermediate position compared to the other groups, although they were relatively distant to each other. The *F. novicida*-infected cultures were localized far away from each other and also far from the SCHU S4-infected cultures, in particular the culture with *GBP* ^chr3^-deficient BMDM (**Figure 2**). The classification correctness of the model built by the linear discriminate analysis was 100% for three groups and 83% for the remaining groups; LVS-infected co-cultures and the *F. novicida*-infected co-culture with *GBP* ^chr3^-deficient BMDM (**Figure 2**).

**Table 7:** Stepwise LDA was used to select variables that best classified the co-cultures according to BMDM phenotype and type of infection.

Step	Stepwise test of variables giving thethe highest performance^1^	Wilks lambda	F-value
1	MCP-1	0.307	30
2	MCP-1+GM-CSF	0.115	58
3	MCP-1+GM-CSF+RANTES	0.037	77.7
4	MCP-1+GM-CSF+RANTES+IL-6	0.014	90.5
5	MCP-1+GM-CSF+RANTES+IL-6+Eotaxin	0.006	98.1
6	GM-CSF+RANTES+IL-6+Eotaxin	0.009	90.5
7	GM-CSF+RANTES+IL-6+Eotaxin+IL-10	0.004	98.1

^1^See section Data Analysis and Statistical Methods for a description of the prediction model.

In summary, using linear discriminant analysis and combining values for five cytokines, a high-resolution identification was achieved for each of the co-culture groups.

## Discussion

Cell-mediated immunity is essential for control of a majority of intracellular microorganisms, however, to qualitatively and quantitatively characterize cell-mediated mechanisms executing protection is very challenging, unlike measurements used to describe humoral immune responses. In fact, there are no validated methods for describing cell-mediated correlates of protection and this is an obvious limitation hampering rational vaccine development for infections caused by intracellular microorganisms. With regard to development of tularemia vaccines, another limitation is the fact that tularemia is an infrequent disease in most countries and in other countries occurring very irregularly, therefore, clinical trials to assess vaccine efficacy will not be feasible. Thus, the Animal Rule may be the only possibility to license a tularemia vaccine in the future. In this context, the co-culture described herein will be one of several models that provide important information, since it in many respects reflects the complexity of cell-mediated immune responses *in vivo* and allows for elaborate measurements of the molecular mechanisms at work.

Previous work based on the use of co-culture methods have identified mechanisms that closely correlate to the degree of protection observed and in this regard we and others have previously demonstrated the critical roles of, *e.g*., nitric oxide, IFN-γ, IL-17, GM-CSF, and TNF in the mouse model (Elkins et al., 2011; de Pascalis et al., 2012; de Pascalis et al., 2014; Golovliov et al., 2016). All of these cytokines are in several infectious model involved in the development of Th1 immunity, which is critical for the successful control of an *F. tularensis* infection. Numerous studies have identified the critical role of IFN-γ for the control of intracellular infection with *F. tularensis* or *F. novicida* (Anthony et al., 1991; Fortier et al., 1992; Polsinelli et al., 1994; Lindgren et al., 2005; Santic et al., 2005; Lindgren et al., 2007; Edwards et al., 2010). However, the IFN-γ-dependent effector mechanisms have long been elusive and numerous IFN-γ-inducible factors with potent bactericidal activities have been excluded as essential, *e.g*., reactive oxygen or nitrogen species, tryptophan degradation, autophagy, and various forms of cell death (Edwards et al., 2010). Our understanding of IFN-γ-dependent effector mechanisms has been greatly advanced with the identification of GBPs and their important role for the control of many intracellular microorganisms (Meunier and Broz, 2016). Not the least *F. novicida* has been a focus of these studies, since it was demonstrated that GBPs are critically required for activation of AIM2 in conjunction with an *F. novicida* infection (Man et al., 2015; Meunier et al., 2015). In addition to previous demonstrations of their critical role for the protection against vacuolar pathogens, *F. novicida* became the first example that GBPs also target cytosolic bacteria (Meunier and Broz, 2016). Notably, subsequently it was demonstrated that the IFN-γ-induced control of *F. novicida* replication also occurred in macrophages deficient for caspase-1 and caspase-11 combined, or for AIM2, and independent of NADPH oxidase and nitric oxide synthase, all of which have been identified as IFN-γ-inducible effectors known to act downstream of GBPs (Wallet et al., 2017). Thereby, these findings identified GBPs as the most critical effector of IFN-γ-mediated killing of *F. novicida*. It was observed that the prerequisites for control of BMDM infection with the LVS strain was similar to that of *F. novicida*, whereas replication of the SCHU S4 strain was not affected by IFN-γ activation (Wallet et al., 2017).

To further understand the protective mechanisms controlling *F. tularensis* and *F. novicida* in the murine model, we believed that implementation of the co-culture model would be of relevance and may provide additional information beyond that of an intracellular infection model based on a single cell-type. Moreover, in contrast to previous publications investigating the role of GBPs, our study assessed their role for acquired immune responses. Previously, we and others have demonstrated that the use of splenocytes from vaccinated animals in the model is highly relevant, since their protective capacity as effector cells closely mimics the efficacy of the vaccination regime in both mice and rats (Golovliov et al., 2016; de Pascalis et al., 2018; Lindgren et al., 2020). Thus, the model can be assumed to closely reflect the complex acquired cell-mediated immune response occurring *in vivo*. In view of the previous findings that the requirements for GBPs to control an *Francisella* infection was distinct between the LVS strain and *F. novicida*, on one hand, and the SCHU S4 strain on the other hand (Wallet et al., 2017), warranted the inclusion of all three strains in the study.

Our present results are in agreement with those of previous studies using the mouse co-culture assay demonstrating correlation between levels of Th1 cytokines, such as IL-2, GM-CSF, IFN-γ, TNF, and MIP-1β and the degree of protection (Golovliov et al., 2016; de Pascalis et al., 2018; Lindgren et al., 2020). Moreover, we have established a human co-culture assay model and identified IFN-γ, TNF, and MIP-1β as protective correlates (Eneslätt et al., 2018). Thus, many of the cytokines identified in the various *in vitro* and *in vivo* model are overlapping and therefore corroborate their relevance for protection. In view of the ability of each of these cytokines to potentiate the ability of macrophages to control intracellular pathogens, the finding is not surprising. The results also demonstrate that control of even highly virulent strains is achievable in the co-culture model, in agreement with previous data (Mahawar et al., 2013; Griffin et al., 2015; Golovliov et al., 2016; Eneslätt et al., 2018; Lindgren et al., 2020), although the degree of control was less marked with the highly virulent SCHU S4 strain as compared to the LVS or *F. novicida* strains. Thus, the strain with the highest virulence, SCHU S4, demonstrated the most rapid replication and also was the least affected by the presence of immune cells, likely due to its potent immunomodulatory properties (Melillo et al., 2010; Gillette et al., 2014; Ireland et al., 2018).

A focus of the present study was to understand the role of GBPs in the adaptive anti-*Francisella* immune defense. The findings unequivocally demonstrate their dominant role for the control of infection, regardless of infectious agent, and in their absence, no significant control was observed. However, in cultures with *GBP* ^chr3^-deficient BMDM, there was consistently lower numbers of all three bacteria in the presence of immune splenocytes *vs*. naïve splenocytes. It cannot be excluded that these differences, although rather small, are indicative of low-level protection in cultures with immune splenocytes and *GBP* ^chr3^-deficient BMDM and that a larger data-set would have corroborated this.

The results in the co-culture mirror to some extent the virulence of the infection agents, in as much as the degree of control of bacterial replication effectuated in the cultures was much more prominent for the low virulent strains *F. novicida* and LVS than for the high virulent strain SCHU S4. Also, the cytokine profiles of the culture supernatants were very much dependent on the infectious agent and in particular, the profiles of the *F. novicida*-infected cultures were distinct from the other cultures. Notably, the cytokine profiles of the SCHU S4 and LVS-infected cultures showed much similarities in cultures with immune splenocytes and wild-type or *GBP* ^chr3^-deficient BMDM, although control of the latter infection was much more marked in cultures with wild-type BMDM. Also, the most marked differences with regard to cytokine profiles were found between cultures with immune *vs*. naïve splenocytes, at least in cultures infected with SCHU S4 and LVS. This implies that the difference between the two infections mostly is dependent on the relative susceptibility of SCHU S4 and LVS to the GBP-mediated killing, rather than the ability of each bacterium to modulate the immune responses. Whereas the SCHU S4 and LVS-infected cultures demonstrated significantly higher levels of a majority of cytokines in immune splenocyte cultures *vs*. naïve splenocyte cultures, this was not the case for the *F. novicida* cultures. For both of the former infections, IFN-γ and GM-CSF were increased in cultures with immune splenocytes, which is of relevance since both have been correlated to protection in other studies on *Francisella*. Our results further demonstrate that the cytokines secreted in the cultures with immune splenocytes were rather independent of GBPs. This is of interest since it has been demonstrated that GBPs serve a very important role for regulation of multiple inflammasomes (Wallet et al., 2017), whereas they in the complex co-culture do not appear to be critically required for cytokine regulation at the time point investigated. In view of their critical role for control of infection, this implicates that the main function for the GBPs in the co-culture system with immune effector cells is to execute a cell-autonomous bactericidal effect.

Few studies have compared the cytokine patterns during infections with bacteria of variable virulence in the co-culture model (Eneslätt et al., 2018), but there are several studies that consistently have reported that the monocytic cells demonstrate much more pronounced inflammatory responses during infection with *F. novicida* than with LVS (Gavrilin et al., 2006; Bröms et al., 2011). In addition, the immunomodulatory abilities of the LVS and SCHU S4 strains have been extensively studied (Telepnev et al., 2005; Gavrilin et al., 2006; McCaffrey and Allen, 2006; Weiss et al., 2007; Bröms et al., 2011; Lindgren et al., 2013; Rabadi et al., 2016). Specifically, a number of potent immunosuppressive traits of SCHU S4 have been identified during infection of monocytic cells (Melillo et al., 2010; Gillette et al., 2014; Ireland et al., 2018). In view of this background, we find it unsurprising that there were distinct cytokine patterns observed in response to infection with each of the three bacterial strains.

Linear discriminant modeling was performed to identify individual cytokines that correlated to control of infection. The modeling revealed that the identified cytokines to some extent was dependent on the infectious agent, but some cytokines were consistently identified to predict the type of infection, *i.e*., MCP-1, Eotaxin, RANTES, GM-CSF, IL-6, and IL-10. Since these cytokines represent a diverse set of functions; chemokines, Th1 cytokines, pro- and anti-inflammatory cytokines, the findings illustrate the complexity of the immune responses elicited in the co-culture model. This data together with data obtained previously using various *in vitro* and *in vivo* models will make it possible to obtain a comprehensive understanding of the protective immune responses in various animal species as well as in different types of tissues against *F. tularensis*.

## Data Availability Statement

The raw data supporting the conclusions of this article will be made available by the authors, without undue reservation.

## Ethics Statement

The animal study was reviewed and approved by Ethical Committee on Animal Research, Umeå, Sweden and the University of Lyon, France (CEC-CAPP).

## Author Contributions

NM, HL, IG, and KE performed all experiments. AM prepared GBP chr3-deficient macrophages. AS, NM, and HL designed the study, analyzed the data, and wrote the manuscript. TH wrote and reviewed the manuscript. MY provided the GBP chr3-deficient mice. All authors contributed to the article and approved the submitted version.

## Funding

Grant support was obtained from Region Västerbotten (Spjutspetsmedel, VLL-582571, Centrala ALF medel, VLL-463691). MY was supported by the Research Program on Emerging and Re-emerging Infectious Diseases (JP20fk0108137), Japanese Initiative for Progress of Research on Infectious Diseases for global Epidemic (JP20wm0325010) and Strategic International Collaborative Research Program (JP19jm0210067) from Agency for Medical Research and Development (AMED), Grant-in-Aid for Scientific Research on Innovative Areas (Production, function and structure of neo-self; 19H04809), for Scientific Research, 18KK0226, 18H02642, and 19H00970, from Ministry of Education, Culture, Sports, Science and Technology of Japan.

## Conflict of Interest

The authors declare that the research was conducted in the absence of any commercial or financial relationships that could be construed as a potential conflict of interest.
