# Supplementary figures and images for: Guanylate-Binding Proteins Are Critical for Effective Control of Francisella tularensis Strains in a Mouse Co-Culture System of Adaptive Immunity
Source: Front Cell Infect Microbiol. 2020 Dec 10;10:594063. doi: 10.3389/fcimb.2020.594063 (PMC7758253; doi:10.3389/fcimb.2020.594063)

## Slide 1
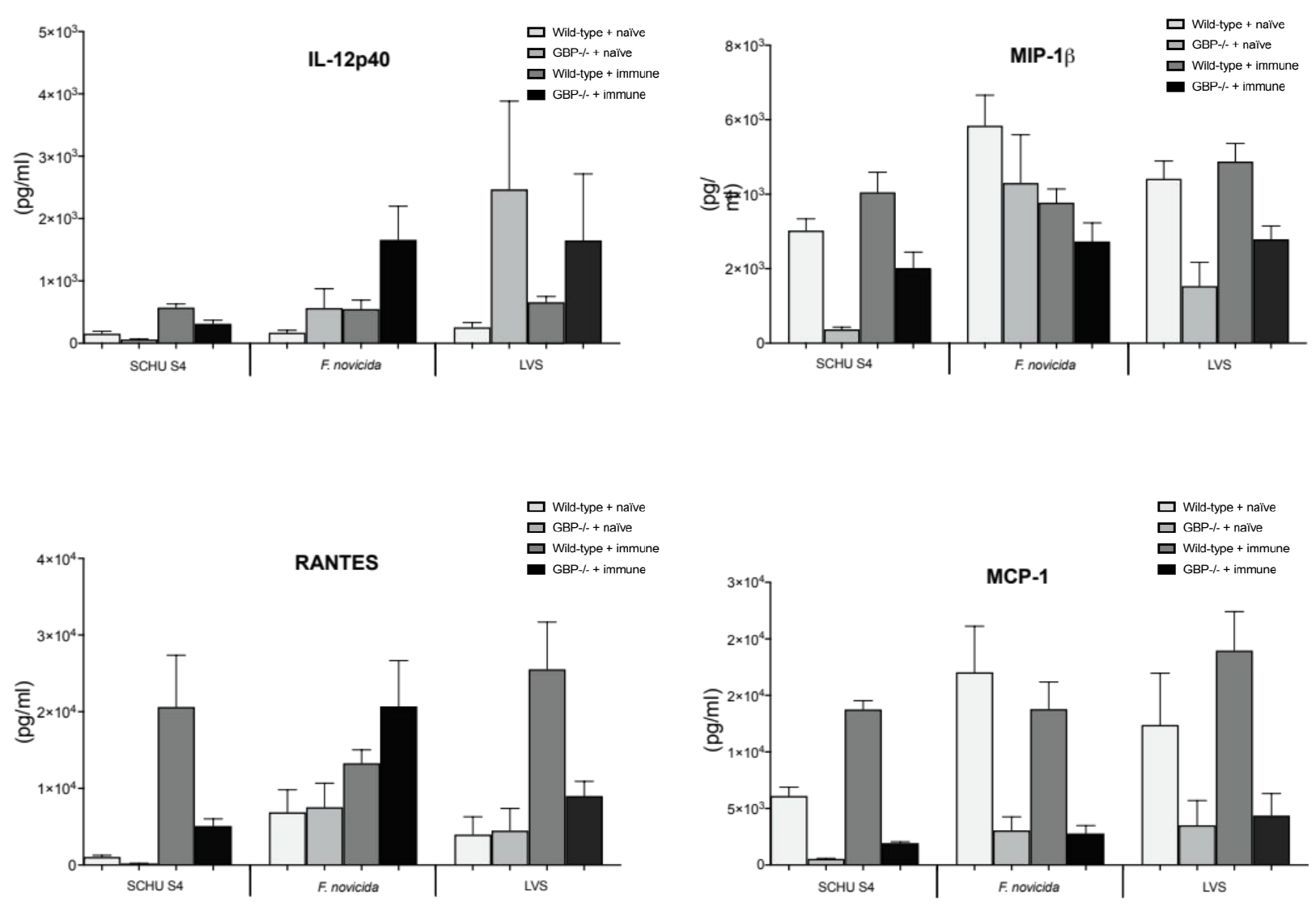

## Slide 2
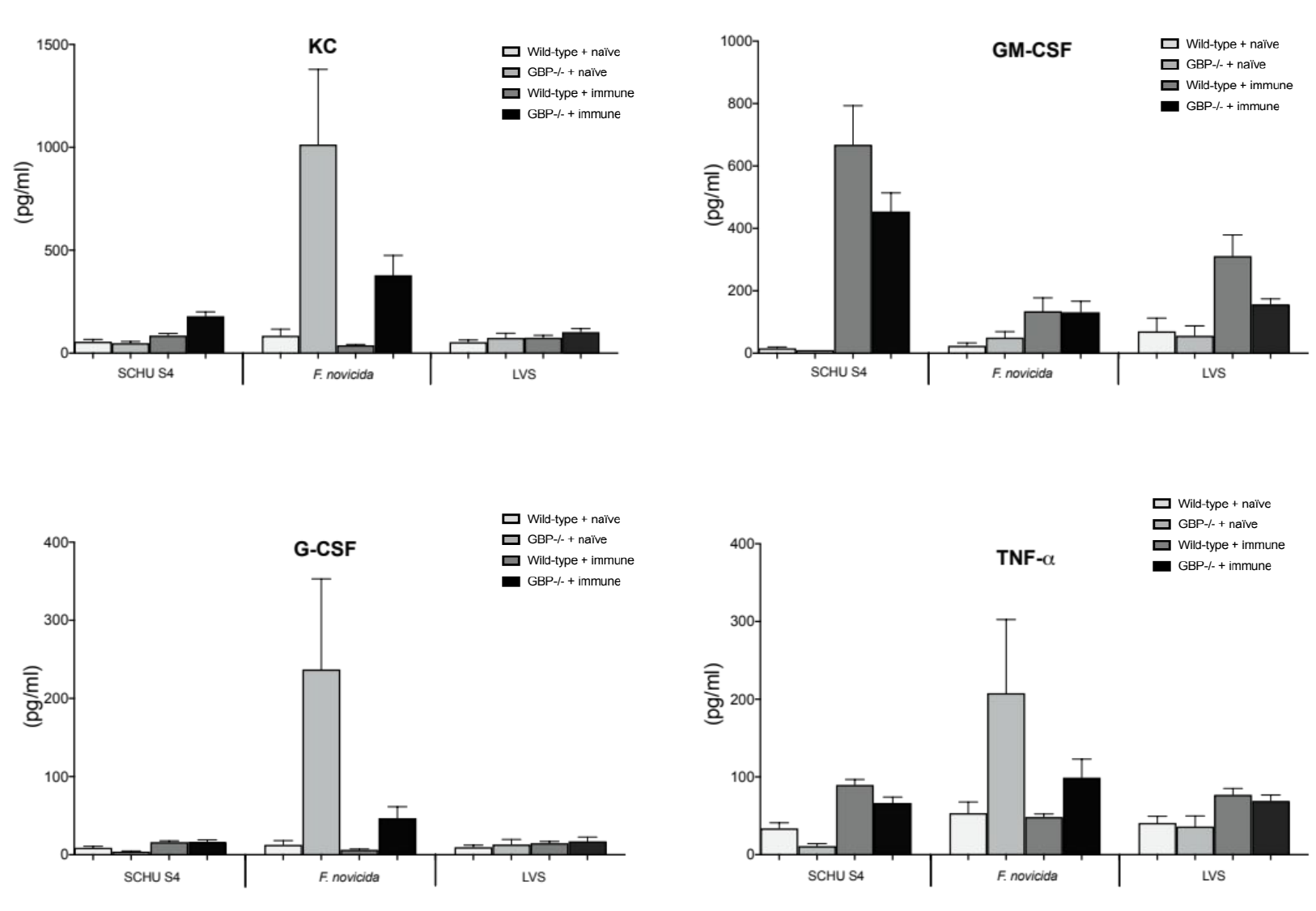

## Slide 3
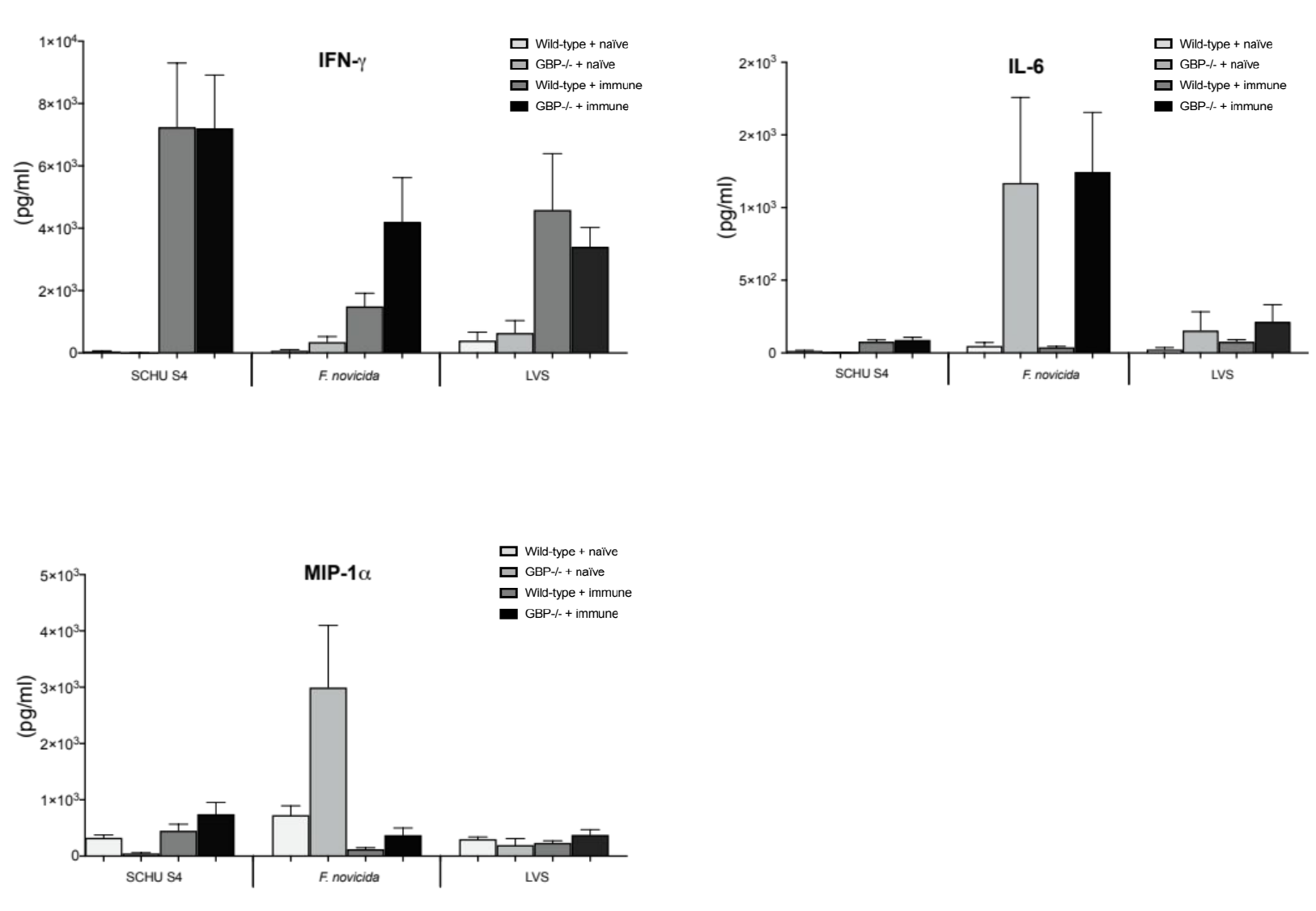

## Slide 4
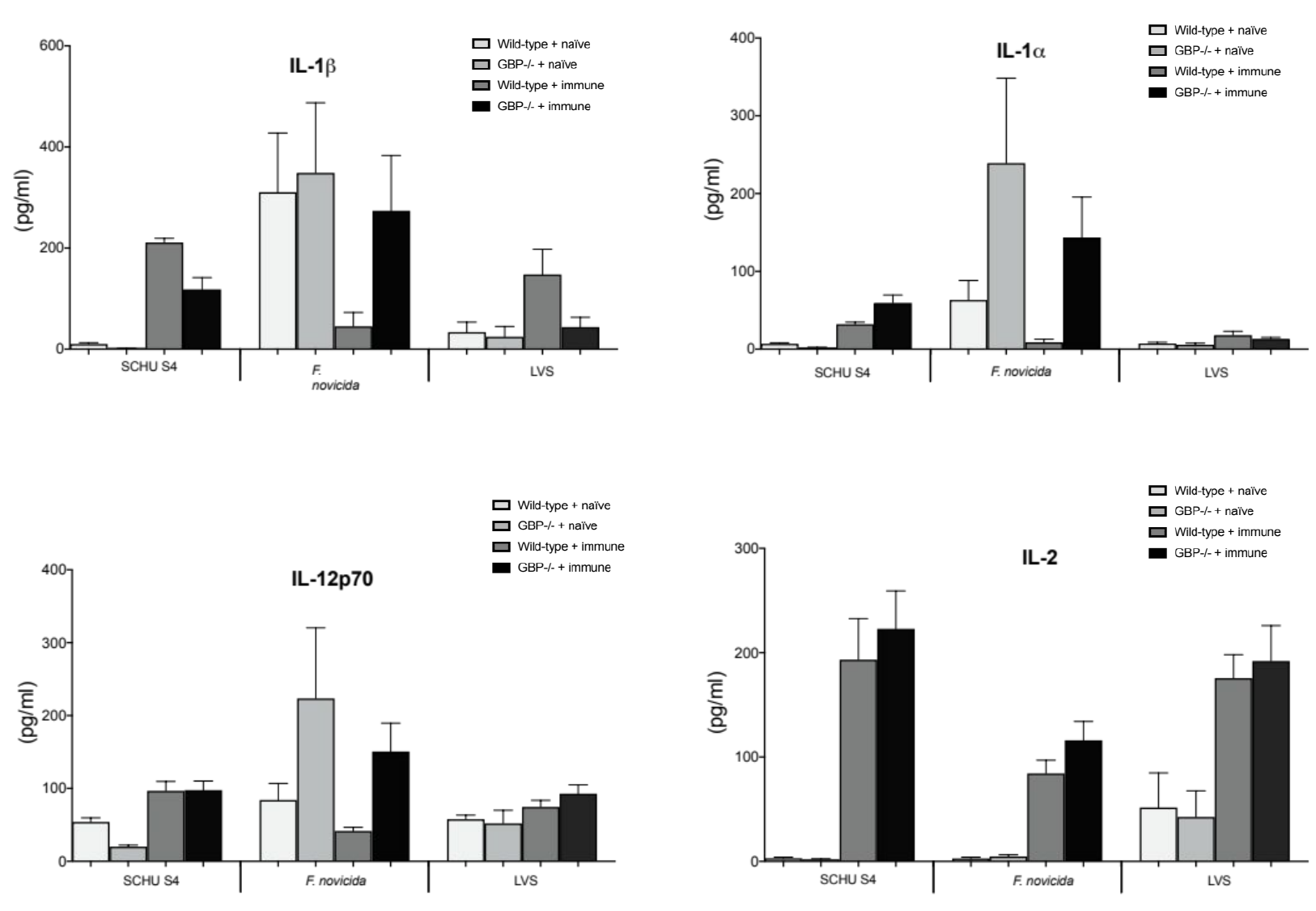

## Slide 5
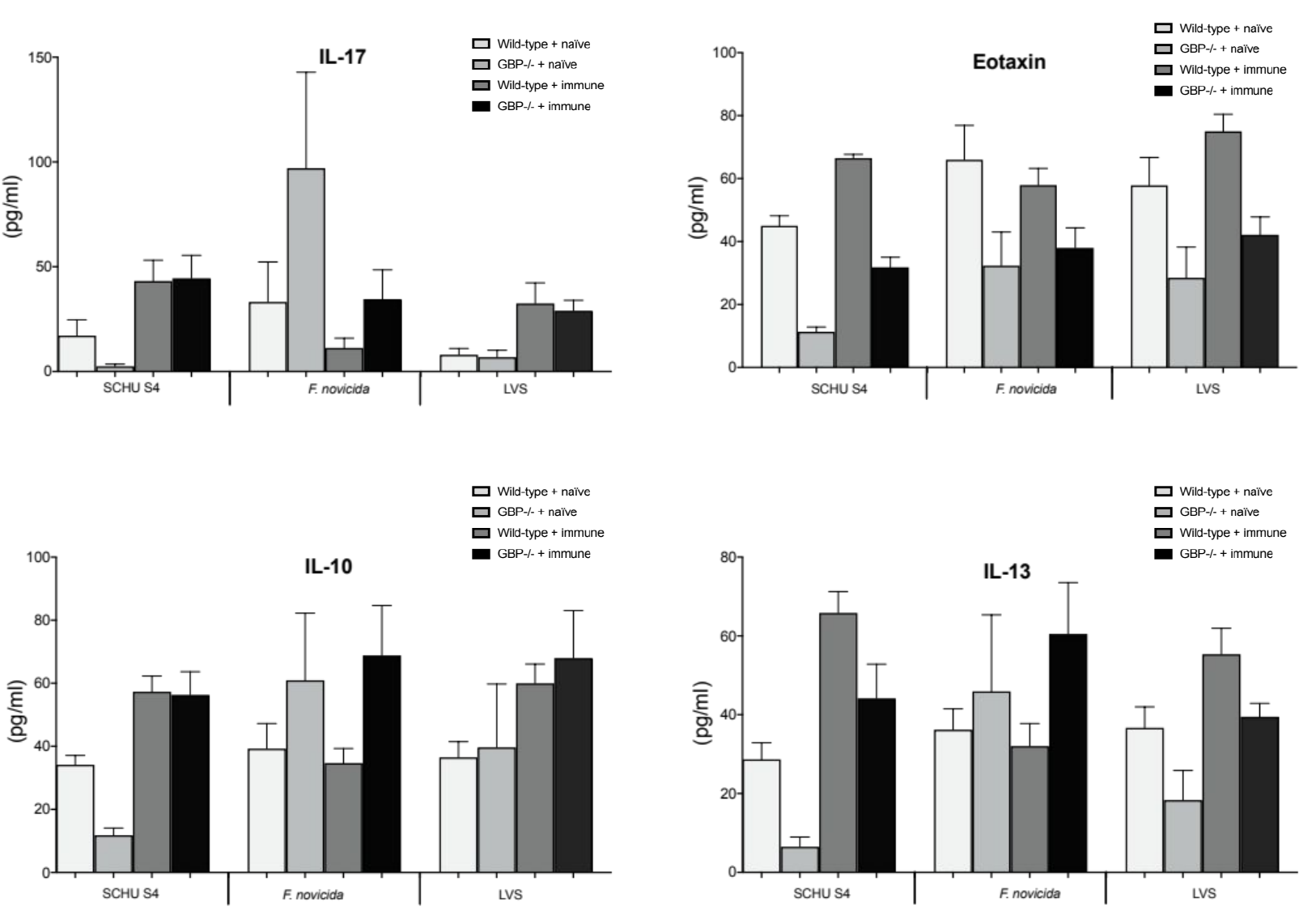

## Slide 6
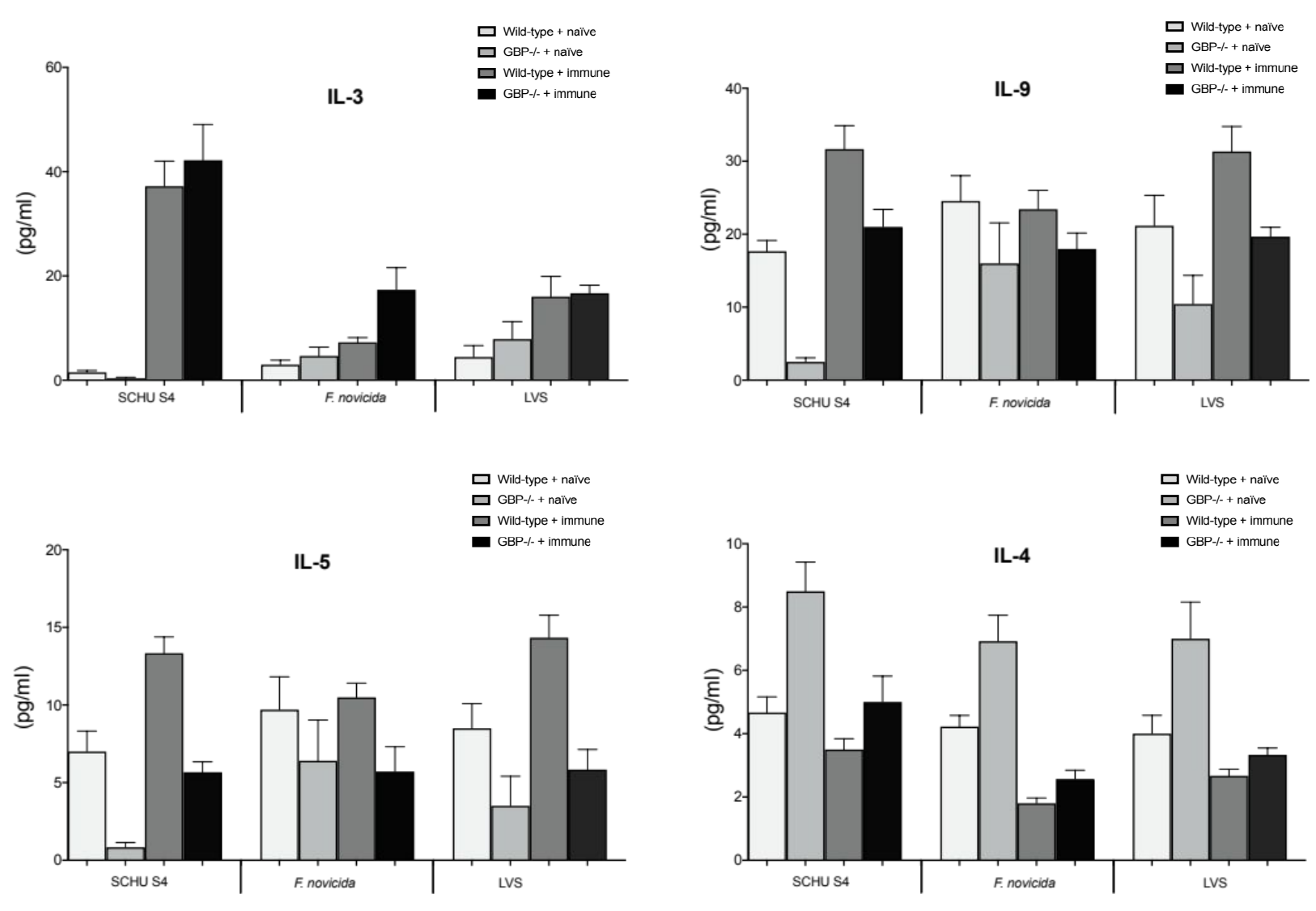

Supplement: Supplementary file 1 [file Presentation_1.pptx]
